# Association of *TGF-ß1* polymorphisms and chronic hepatitis C infection: a Meta-analysis

**DOI:** 10.1186/s12879-019-4390-8

**Published:** 2019-08-30

**Authors:** Pengfei Guo, Shuangyin Liu, Xiangru Sun, Longqin Xu

**Affiliations:** 1 College of Computational Science, Zhongkai University of Agriculture and Engineering, Guangzhou, 510200 China; 2 College of Information Science and Technology, Zhongkai University of Agriculture and Engineering, Guangzhou, 510200 China; 3 Intelligent Agriculture Engineering Research Center of Guangdong Higher Education Institutes, Zhongkai University of Agriculture and Engineering, Guangzhou, 510200 China; 4 Guangdong Province Key Laboratory of Waterfowl Healthy Breeding, Zhongkai University of Agriculture and Engineering, Guangzhou, 510200 China; 5 Institute of Reproductive Medicine, Affiliated Hexian Memorial Hospital, Southern Medical University, Guangzhou, 511400 China

**Keywords:** Single nucleotide polymorphism, *TGF-ß1* gene, Quality assessment, Meta-analysis, Hepatitis C virus

## Abstract

**Background:**

Although several researches have reported the connection between the transforming growth factor-beta 1 (*TGF-β1*) gene polymorphisms and chronic hepatitis C virus (HCV) infection, the conclusions of these studies were not always consistent. Here, this paper proposed a meta-analysis to evaluate whether the *TGF-ß1* gene polymorphisms, −509C/T (rs1800469), codon 10 T/C (rs1982073) and codon 25G/C (rs1800471), were associated with chronic HCV infection.

**Methods:**

The summary odds ratios (ORs) of chronic HCV infected patients and controls with all SNPs were obtained by adaptive fixed or random effect model. A series of statistical tools were employed to guarantee the accuracy of related pooling ORs, including the Hardy-Weinberg equilibrium (HWE) test, sensitivity analysis and publication bias test.

**Results:**

This paper analyzed 18 case-control studies in 17 articles which totally contains 2718 chronic HCV infection cases corresponding to 1964 controls. The results of the meta-analysis indicated that the −509C/T polymorphism effected an increased risk of chronic HCV infection in all gene models. More specifically by ethnicity stratification, the Egyptians shared the similar association with the above overall study. Moreover, the meta-fusion of healthy control studies showed that − 509 T allele carriers (TT + TA) had nearly 2.00 and 3.36 fold higher risk of chronic HCV infection in the total and Egyptian populations, respectively (OR = 2.004, 95% CI = 1.138–3.528, *P* = 0.016; OR = 3.363, 95% CI = 1.477–7.655, *P* = 0.004, respectively). However, our meta-analysis did not find any significant association between the codon 10 T/C or codon 25G/C polymorphisms and chronic HCV infection.

**Conclusions:**

Our results suggested that the *TGF-ß1*–509C/T polymorphism may effect an increased risk of chronic HCV infection, especially in Egyptian population.

**Electronic supplementary material:**

The online version of this article (10.1186/s12879-019-4390-8) contains supplementary material, which is available to authorized users.

## Background

Hepatitis C virus (HCV) infection which is a widely prevalent infectious disease has presented in about 170 million people of the world [1]. There are 60–80% of patients with the acute infection developing into chronic hepatitis C (CHC). In the long run, one out of three CHC patients will progress to hepatic complications such as hepatic fibrosis, liver cirrhosis (LC), eventually hepatocellular carcinoma (HCC), which leads to high mortality [2]. For a long time, scientists have revealed the factors which regulate the responses to HCV infection and affect disease progression. Some studies reported that the viral genotypes, environmental factors and behavioral factors (excessive alcohol intake) were implicated in the development of HCV infection [3, 4]. However, these factors cannot fully explain the large variability in susceptibility or outcomes observed within different populations. Recently, several genetic association studies concluded that the mutations of certain cytokine genes may play an important role in the susceptibility and progression of HCV infection, due to the insufficient or imbalance responses in the cytokine network [5–7].

Transforming growth factor-beta 1 (TGF-β1), which is an crucial immuno-regulatory cytokine secreted by hepatic stellate cells, fibroblasts, and Kupffer cells, is participated in the regulation of cellular growth, differentiation and proliferation [8]. During the acute infectious stage of HCV, natural killer (NK) cells produce interferon-gamma (IFN-γ), and the proliferation and cytotoxicity of NK cells are pivotal in clearing HCV infection. As a renowned suppressor of NK cells, TGF-β1 inhibits the secretion of IFN-γ and interleukin (IL) -12, leading to the persistence of HCV infection [6]. In different infection states, the frequent mutations and expression of *TGF-β1* were various implying a possible role of TGF-β1 in HCV infection [6, 9]. The *TGF-β1* gene which is located in chromosome 19q13.1 is constituted by 7 exons and 6 introns. To date, several functional single-nucleotide polymorphisms (SNPs) of *TGF-β1* have been reported. Particularly, the −509C/T (rs1800469), codon 25G/C (rs1800471), and codon 10 T/C (rs1982073) SNPs are the most widely evaluated polymorphisms [10, 11]. It has been demonstrated that these functional SNPs are associated with the interindividual differences of *TGF-β1* expression [6, 7]. The above facts suggest that −509C/T, codon 25G/C, and codon 10 T/C SNPs may contribute to TGF-β1-mediated immune response in HCV infection.

Recently, great attention has paid to investigate whether the −509C/T, codon 25G/C, and codon 10 T/C SNPs of *TGF-β1* gene were associated with the chronic HCV infection. Pooling the related research’s data, we found that the conclusions of these studies were not always consistent. Taking the *TGF-β1*–509C/T polymorphism as an example, most studies suggested that people with the −509TT genotype and T allele have a higher risk of chronic HCV infection [6, 7, 12, 13]; and Kimura et al. described −509CC genotype and C allele may contribute to HCV clearance rates in Japanese populations [14]; however, no association has been shown in another study [15]. These reported discrepancies may result from the differences of individual studies in sample size, as well as geographical region and ethnicity of the subjects.

In this paper, we proposed a meta-analysis by pooling the small size case-control results statistically to further clarify the role of *TGF-ß1*–509C/T (rs1800469), codon 10 T/C (rs1982073), and codon 25G/C (rs1800471) polymorphisms in chronic HCV infection in order to overcome the drawbacks of unbalance sampling data-driven experiment result.

## Methods

### Identification of eligible studies

The genetic association studies which was adopted by this paper published before May 2019. The adopted articles of *TGF-ß1* gene polymorphisms and HCV infection were sought in PubMed, Web of Science and EMBASE (Excerpta Medica Data base). The keywords of search were used as follow: (Transforming Growth Factor-beta OR *TGF-ß1*) AND (Polymorphism OR SNP) AND (HCV infection OR clearance), without language restriction. Additionally, we searched the other related review articles and the references of articles by hand identifying.

### Inclusion and exclusion criteria

Defining an article as eligible study if: (i) The article conducted an evaluation of the association of *TGF-ß1* gene polymorphisms (−509C/T, codon 25G/C and codon 10 T/C) with the spontaneous clearance of HCV or the susceptibility of chronic HCV infection; (ii) The article was a case-control or cohort study; (iii) The article provided enough subjects data to calculate the odds ratios (ORs) of association with 95% confidence intervals (CIs). One study will be excluded if the patients were reported as co-infected (infected) with the other virus (human immune deficiency virus, HIV or HBV) or the liver transplant recipients. For the overlapping studies, we selected the most recent or complete publication.

### Definitions

The definition of asymptomatic carriers (AS) of HCV is as follow: (i) The patients have been infected persistently with HCV; (ii) The patients don’t display sign/symptom; (iii) There exists necro-inflammatory cells.

CHC can be described as follow: (i) CHC is a chronic neuroinflammatory disease of the liver; (ii) The index of anti-HCV antibodies is positive; (iii) The index of HCV RNA is positive. Usually, CHC is caused by the persistent infection with HCV.

The spontaneously recovered (SR) subjects of HCV infection can be defined by the following conditions: (i) The index of anti-HCV antibodies is positive; (ii) The index of HCV RNA is negative; (iii) The function tests of liver are normal; (iv). No history of HCV vaccination. In this paper, we discussed the problem of chronic HCV infection which consisted of CHC, AS and liver cirrhosis.

### Data extraction

Information was extracted from these included studies by two of the authors independently (Guo PF and Sun XR). We extracted the following detail information from the included studies: the name of the first author, the year of publication, the ethnicity, the geographic location, genotyping method, definitions and total amount of cases and controls, frequency of genotypes. The control groups were further divided into the SR and healthy control groups.

### Quality assessment

In order to make our results more credible, we conducted a quality assessment of the included studies according to the scale of the properties of the piece study data (details showed in Additional file 4: Table S4). Five items were assessed in this scale, including the representation of the cases, the source of controls, the number of samples, the genotyping method’s quality control and the Hardy-Weinberg equilibrium (HWE). The quality of studies was scored by integrals which was ranged in interval (0–10). The lower scored studies have been dropped by careful discussion. The results of quality assessment were shown in Table 1.

**Table 1:** Characteristics of studies evaluating the effects of TGF-ß1 gene polymorphisms on chronic HCV infection of the Meta-Analysis

First author	Year	Country	Ethnicity	QA	Genotyping	Case		Control		Position
[Reference]					method	Sample	Numbers	Sample	Numbers	
Larijani [16]	2016	Iran	Caucasian	7	PCR-AS	CHC	89	Healthy	76	-509
Ma [13]	2015	China	Asian	6	PCR-RFLP	CHC	393	Healthy	375	-509
Imran [17]	2014	Pakistan	Asian	7	PCR-AS	CHC	140	Healthy	120	codon 10,25
Mohy [6]	2014	Egypt	Egyptian	4	PCR-RFLP	LC	40	Healthy	40	-509
Rebbani [18]	2014	Morocco	Mix	8	PCR-RFLP	CHC	119	Healthy	137	codon 25
								SR	54	codon 25
Pasha [14]	2013	Egypt	Egyptian	9	PCR-SSP	CHC	440	Healthy	220	-509
Radwan [7]	2012	Egypt	Egyptian	9	PCR-RFLP	CHC	280	Healthy	160	-509
Romani [15]	2011	Iran	Caucasian	9	PCR-RFLP	CHC	164	Healthy	169	codon 10,25,-509
Pereira [19]	2008	Brazil	Mix	7	PCR-SSP	CHC	128	Healthy	94	codon 10,25
Armenda’riz-Borunda [20]	2008	Moxico	Mix	6	PCR-AS	LC	13	Healthy	30	codon 25
Fang [21]	2008	China	Asian	6	PCR-ARMS	CHC	85	Healthy	106	codon 25
Wang [22]	2005	Germany	Caucasian	7	DS	CHC	210	Healthy	50	codon 10,25
Kimura [23]	2005	Japan	Asian	6	PCR-AS	CHC	184	SR	46	-509
Zein [24]	2004	Egypt	Egyptian	8	DS	CHC	24	Healthy	47	codon 10,25
		USA	Caucasian	8	DS	CHC	31	Healthy	36	codon 10,25
Suzuki [25]	2003	Japan	Asian	8	PCR-RFLP	CHC	206	Healthy	101	codon 10
Barrett [26]	2003	Ireland	Caucasian	5	PCR-SSP	CHC	92	SR	66	codon 10,25
Vidigal [27]	2002	Brazil	Mix	8	DS	CHC	80	Healthy	37	codon 10,25

### Statistical analysis

In this paper, we quantified the relationship between the three crucial SNPs of *TGF-ß1* and chronic HCV infection by pooling the ORs with its 95% CI. For the purpose of obtaining the role of these SNPs in chronic HCV infection, we used the following five gene models to extract the related statistical information: the homozygote model, the heterozygous model, the dominant comparison model, the recessive model, and the allele contrast model.

For each included study, the χ^2^ test is used to measure the HWE. The HWE is said to be significant if the estimator of the χ^2^ test is larger than 0.05. The heterogeneity of between-studies can be quantified by the Q-statistic. Additionally, the *I*^*2*^ statistic measures the degree of heterogeneity [28]. By the indexes of the above statistical qualification, we selected the effect model as following rules: (1) If the *p*-value is less than 0.10 or the value of *I*^*2*^ is more than 50%, then the effects are inconsistent, and the random-effects model will be selected; (2) If the *p*-value and the value of *I*^*2*^ are belonging to the exception of condition (1), then the effects are consistent, the fixed-effects model will be selected. In order to evaluate the pooling results, the Z-test is used for assessing the significance of the summary ORs.

We obtained the specific information of different ethnicities by exploring the sources of heterogeneity which is conducted by stratified analysis. Moreover, the robustness of the summary results can be assessed through sensitivity analysis. In addition, the potential publication bias of studies can be quantified by the Begg’s funnel plot and the Egger’s test [29].

These statistical analyses mentioned above were implemented on Stata 11.0 software.

## Results

### Studies included in the meta-analysis

**Fig. 1:**
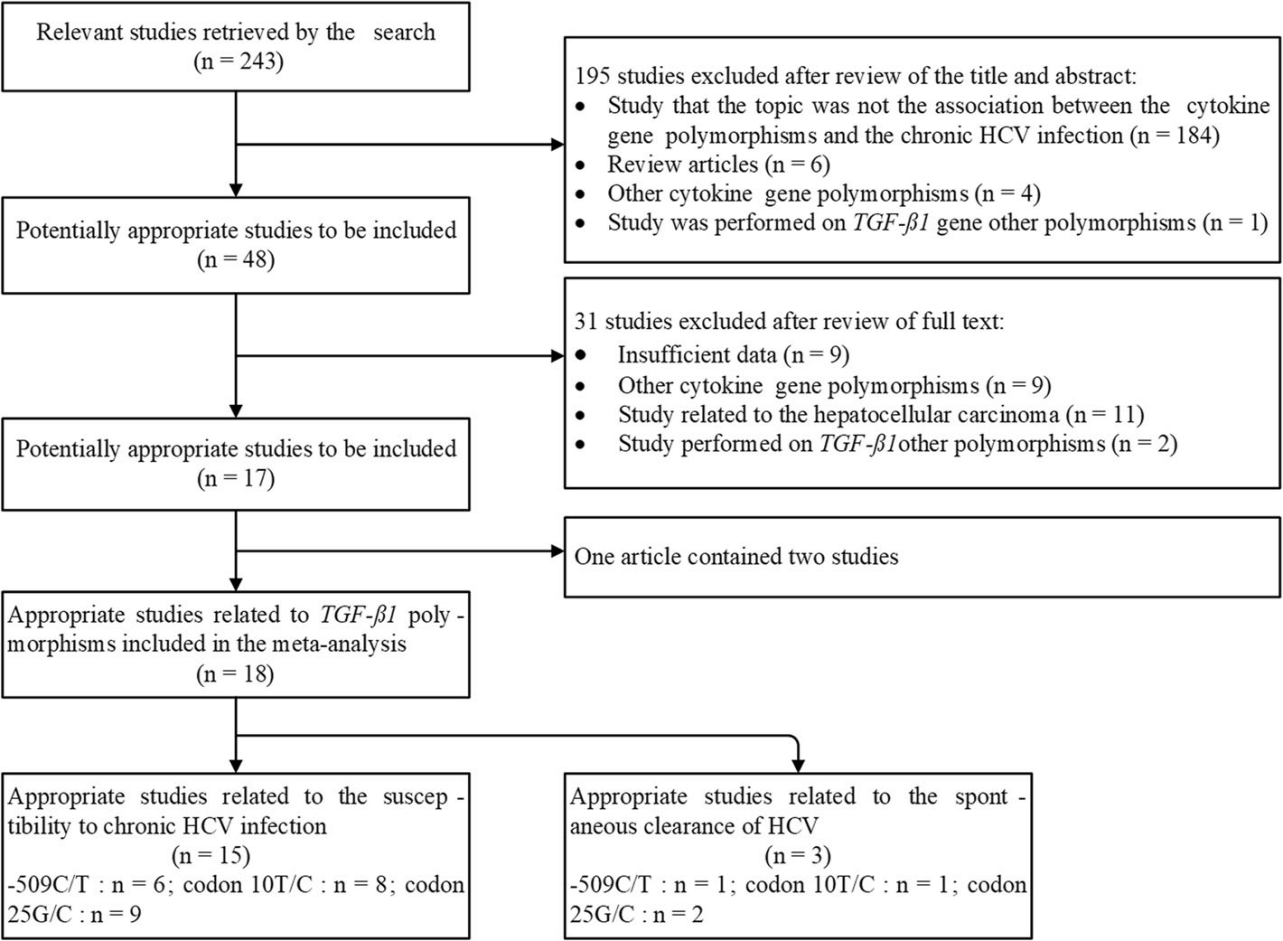
Flow chart of study selection

The process of the included studies selection was shown in the flowchart (Fig.1). One article was regarded as two separated studies as it contained two independent case-control studies [30]. Finally, a total of 18 case-control (separated) studies were selected to conduct meta-analysis. All the selected studies have designed experiments to reveal the connections of *TGF-ß1*–509C/T, codon 10 T/C, and codon 25G/C polymorphisms with the susceptibility to chronic HCV infection or spontaneous clearance of HCV [6, 7, 12–24, 26, 30]. These studies contain 2718 chronic HCV infection cases which correspond to 1964 controls. In the process of meta-analysis, the control subjects were composed of healthy populations [6, 7, 12–16, 18–22, 26, 30] and SR populations [16, 17, 23]. The gene distribution of control groups in two studies for −509C/T polymorphism was deviated from HWE [6, 12]. The stratified analysis was conducted by dividing the eligible 18 studies into the following partitions: Caucasian population (5) ˅ Asian population (5) ˅ Egyptian population (4) ˅ mixed population (4). The main detail information of each included study was summarized in Table 1. Moreover, we extracted the explicit genotype distribution of the three SNPs which were described in Additional file 1: Table S1, Additional file 2: Table S2, Additional file 3: Table S3, respectively.

### Meta-analysis results

As the above mentioned, the total controls were composed of the healthy controls and SR controls. The total controls and chronic HCV infection cases were compared to obtain the generally connection of the three crucial SNPs and chronic HCV infection risk (Table 2). Different controls revealed different relationships between the three crucial SNPs and the infection/clearance of HCV. Specifically, the comparison results of the chronic HCV infected subjects with the healthy control subjects may find the connection of the three crucial SNPs and the susceptibility to chronic HCV infection (Table 3). And the comparison results of the chronic HCV infected subjects with SR control subjects may discover the connection of the three crucial SNPs and the spontaneous clearance of HCV.

**Table 2:** Main results of the meta-analysis of TGF-ß1 gene polymorphisms with the chronic HCV infection in total population

SNPs		No.of study	Gene model	OR(95%CI)	*P*	Heterogeneity		Publication	
						text		bias	
						*P*(Q-test)	*I*^2^(%)	Begg’s	Egger’s
-509	Total	7	**TT vs. CC**	**2.081 (1.249–3.466)**	**0.005**	**0.002**	**73.0**	**0.133**	**0.897**
			**TC vs. CC**	**1.880 (1.162–3.044)**	**0.010**	**0.000**	**79.3**	**0.133**	**0.427**
			**TT + TC vs. CC**	**2.042 (1.240–3.361)**	**0.005**	**0.000**	**83.7**	**0.133**	**0.529**
			**TT vs. CC + TC**	**1.310 (1.114–1.542)**	**0.001**	**0.208**	**30.3**	**0.260**	**0.918**
			**T vs. C**	**1.503 (1.126–2.006)**	**0.006**	**0.000**	**81.8**	**0.230**	**0.580**
	Asian	2	TT vs. CC	1.242 (0.983–1.569)	0.070	0.108	61.2	1.000	–
			TC vs. CC	1.074 (0.939–1.227)	0.298	0.163	48.5	1.000	–
			TT + TC vs. CC	1.077 (0.976–1.190)	0.140	0.102	62.6	1.000	–
			TT vs. CC + TC	1.222 (0.942–1.585)	0.131	0.234	29.4	1.000	–
			T vs. C	1.375 (0.823–2.297)	0.223	0.041	76.1	1.000	–
	Caucasian (Iran)	2	T vs. C	1.007 (0.891–1.139)	0.907	0.584	0.0	1.000	–
	Egyptian	3	TT vs. CC	**3.060 (1.529–6.122)**	**0.002**	**0.046**	**67.5**	**0.296**	**0.895**
			TC vs. CC	**3.123 (1.339–7.284)**	**0.008**	**0.002**	**84.2**	**0.296**	**0.080**
			TT + TC vs. CC	**3.363 (1.477–7.655)**	**0.004**	**0.001**	**86.1**	**0.296**	**0.168**
			TT vs. CC + TC	**1.462 (1.169–1.829)**	**0.001**	**0.260**	**25.8**	**0.296**	**0.981**
			T vs. C	**2.276 (1.295–4.001)**	**0.004**	**0.000**	**87.0**	**0.296**	**0.247**
codon 10	Total	9	CC vs. TT	0.961 (0.802–1.151)	0.664	0.473	0.0	1.000	0.880
codon 25			CT vs. TT	0.961 (0.884–1.045)	0.350	0.975	0.0	0.917	0.301
			CC + CT vs. TT	0.972 (0.911–1.037)	0.390	0.987	0.0	0.917	0.726
			CC vs. TT + CT	1.011 (0.823–1.241)	0.917	0.179	30.0	0.917	0.676
			C vs. T	0.980 (0.907–1.060)	0.613	0.648	0.0	1.000	0.671
	Asian	2	CC vs. TT	1.074 (0.801–1.441)	0.632	0.170	46.8	1.000	–
			CT vs. TT	1.929 (0.804–1.073)	0.318	0.935	0.0	1.000	–
			CC + CT vs. TT	0.973 (0.873–1.085)	0.624	0.514	0.0	1.000	–
			CC vs. TT + CT	1.245 (0.886–1.748)	0.206	0.107	61.5	1.000	–
			C vs. T	1.029 (0.903–1.174)	0.666	0.138	54.5	1.000	–
	Caucasian	4	CC vs. TT	0.950 (0.703–1.284)	0.737	0.350	8.6	1.000	0.976
			CT vs. TT	1.000 (0.876–1.142)	1.000	0.891	0.0	0.734	0.818
			CC + CT vs. TT	0.991 (0.891–1.101)	0.859	0.930	0.0	1.000	0.448
			CC vs. TT + CT	0.934 (0.668–1.305)	0.689	0.220	32.1	1.000	0.861
			C vs. T	0.979 (0.865–1.109)	0.740	0.605	0.0	1.000	0.844
	Mix (Brazilian)	2	CC vs. TT	0.853 (0.587–1.241)	0.406	0.370	0.0	1.000	–
			CT vs. TT	0.904 (0.756–1.082)	0.270	0.916	0.0	1.000	–
			CC + CT vs. TT	0.924 (0.806–1.058)	0.253	0.851	0.0	1.000	–
			CC vs. TT + CT	0.912 (0.588–1.417)	0.683	0.279	14.6	1.000	–
			C vs. T	0.921 (0.779–1.090)	0.338	0.477	0.0	1.000	–
	Total	11	CC vs. GG	0.744 (0.455–1.218)	0.240	0.811	0.0	0.764	0.984
			CG vs. GG	0.940 (0.567–1.559)	0.811	0.003	63.7	0.592	0.748
			CC + CG vs. GG	0.935 (0.570–1.534)	0.790	0.003	64.6	0.592	0.740
			CC vs. GG + CG	0.809 (0.483–1.356)	0.421	0.931	0.0	0.548	0.879
			C vs. G	1.000 (0.670–1.494)	1.000	0.006	59.6	0.436	0.662
	Asian	2	CC vs. GG	0.917 (0.530–1.588)	0.758	0.714	0.0	1.000	–
			CG vs. GG	0.896 (0.680–1.181)	0.435	0.607	0.0	1.000	–
			CC + CG vs. GG	0.917 (0.732–1.148)	0.449	0.737	0.0	1.000	–
			CC vs. GG + CG	0.951 (0.536–1.687)	0.863	0.712	0.0	1.000	–
			C vs. G	0.923 (0.733–1.163)	0.498	0.899	0.0	1.000	–
	Caucasian	5	CC vs. GG	0.274 (0.063–1.190)	0.084	0.513	0.0	1.000	0.880
			CG vs. GG	1.215 (0.563–2.623)	0.620	0.042	59.7	0.806	0.333
			CC + CG vs. GG	1.130 (0.500–2.553)	0.769	0.023	64.8	0.806	0.349
			CC vs. GG + CG	0.339 (0.072–1.600)	0.172	0.767	0.0	1.000	0.882
			C vs. G	1.079 (0.518–2.247)	0.839	0.031	62.4	0.462	0.339
	Mix (Brazilian)	2	CC vs. GG	0.890 (0.117–6.743)	0.910	0.651	0.0	1.000	–
			CG vs. GG	0.648 (0.101–4.170)	0.648	0.014	83.6	1.000	–
			CC + CG vs. GG	0.691 (0.102–4.690)	0.705	0.010	84.8	1.000	–
			CC vs. GG + CG	0.984 (0.126–7.706)	0.988	0.762	0.0	1.000	–
			C vs. G	0.769 (0.129–4.591)	0.774	0.012	84.0	1.000	–
	Mix	3	C vs. G	1.038 (0.272–3.965)	0.956	0.003	83.3	1.000	0.205

A random effects model was used when *P*-value for heterogeneity test was < 0.1; otherwise, a fixed effects model was used, and values in bold were statistically significant at *P* < 0.05. –, no number

*CI* confidence interval, *OR* odds ratio, *SNP* single-nucleotide polymorphism, *P* (Q-test), *P*-value of Q-test for heterogeneity test

**Table 3:** Main results of the meta-analysis of TGF-ß1 gene polymorphisms with the susceptibility to CHC compared with HL population

SNPs		No.of study	Gene model	OR(95%CI)	*P*	Heterogeneity text		Publication bias	
						*P*(Q-test)	*I*^2^(%)	Begg’s	Egger’s
-509	Total	6	TT vs. CC	**1.946 (1.109–3.415)**	**0.020**	**0.002**	**76.2**	**0.221**	**0.949**
			TC vs. CC	**1.878 (1.082–3.261)**	**0.025**	**0.000**	**82.9**	**0.221**	**0.453**
			TT + TC vs. CC	**2.004 (1.138–3.528)**	**0.016**	**0.000**	**86.4**	**0.221**	**0.566**
			TT vs. CC + TC	**1.282 (1.084–1.516)**	**0.004**	**0.177**	**36.7**	**0.462**	**0.862**
			T vs. C	**1.460 (1.065–2.000)**	**0.019**	**0.000**	**83.9**	**0.452**	**0.644**
	Egyptian	3	TT vs. CC	**3.060 (1.529–6.122)**	**0.002**	**0.046**	**67.5**	**0.296**	**0.895**
			TC vs. CC	**3.123 (1.339–7.284)**	**0.008**	**0.002**	**84.2**	**0.296**	**0.080**
			TT + TC vs. CC	**3.363 (1.477–7.655)**	**0.004**	**0.001**	**86.1**	**0.296**	**0.168**
			TT vs. CC + TC	**1.462 (1.169–1.829)**	**0.001**	**0.260**	**25.8**	**0.296**	**0.981**
			T vs. C	**2.276 (1.295–4.001)**	**0.004**	**0.000**	**87.0**	**0.296**	**0.247**
	Caucasian (Iran)	2	T vs. C	1.007 (0.891–1.139)	0.907	0.584	0.0	1.000	–
codon 10	Total	8	CC vs. TT	1.009 (0.837–1.216)	0.926	0.669	0.0	0.902	0.944
			CT vs. TT	0.953 (0.874–1.038)	0.269	0.969	0.0	0.711	0.472
			CC + CT vs. TT	0.974 (0.911–1.041)	0.437	0.972	0.0	1.000	0.701
			CC vs. TT + CT	1.078 (0.870–1.336)	0.492	0.407	3.0	0.902	0.769
			C vs. T	0.995 (0.918–1.079)	0.907	0.725	0.0	1.000	0.811
	Asian	2	CC vs. TT	1.074 (0.801–1.441)	0.632	0.170	46.8	1.000	–
			CT vs. TT	1.929 (0.804–1.073)	0.318	0.935	0.0	1.000	–
			CC + CT vs. TT	0.973 (0.873–1.085)	0.624	0.514	0.0	1.000	–
			CC vs. TT + CT	1.245 (0.886–1.748)	0.206	0.107	61.5	1.000	–
			C vs. T	1.029 (0.903–1.174)	0.666	0.138	54.5	1.000	–
	Caucasian	3	CC vs. TT	1.098 (0.786–1.534)	0.585	0.882	0.0	0.296	0.296
			CT vs. TT	0.987 (0.855–1.139)	0.854	0.784	0.0	1.000	0.682
			CC + CT vs. TT	1.000 (0.892–1.122)	0.995	0.859	0.0	1.000	0.661
			CC vs. TT + CT	1.114 (0.764–1.626)	0.575	0.779	0.0	0.296	0.103
			C vs. T	1.022 (0.890–1.173)	0.763	0.956	0.0	1.000	0.942
	Mix (Brazilian)	2	CC vs. TT	0.853 (0.587–1.241)	0.406	0.370	0.0	1.000	–
			CT vs. TT	0.904 (0.756–1.082)	0.270	0.916	0.0	1.000	–
			CC + CT vs. TT	0.924 (0.806–1.058)	0.253	0.851	0.0	1.000	–
			CC vs. TT + CT	0.912 (0.588–1.417)	0.683	0.279	14.6	1.000	–
			C vs. T	0.921 (0.779–1.090)	0.338	0.477	0.0	1.000	–
codon 25	Total	9	CC vs. GG	0.740 (0.450–1.219)	0.237	0.702	0.0	0.452	0.908
			CG vs. GG	0.832 (0.501–1.383)	0.479	0.012	59.2	0.754	0.693
			CC + CG vs. GG	0.835 (0.505–1.382)	0.484	0.008	61.3	0.754	0.713
			CC vs. GG + CG	0.814 (0.482–1.373)	0.440	0.869	0.0	0.707	0.872
			C vs. G	0.933 (0.612–1.422)	0.747	0.010	58.7	0.371	0.634
	Asian	2	CC vs. GG	0.917 (0.530–1.588)	0.758	0.714	0.0	1.000	–
			CG vs. GG	0.896 (0.680–1.181)	0.435	0.607	0.0	1.000	–
			CC + CG vs. GG	0.917 (0.732–1.148)	0.449	0.737	0.0	1.000	–
			CC vs. GG + CG	0.951 (0.536–1.687)	0.863	0.712	0.0	1.000	–
			C vs. G	0.923 (0.733–1.163)	0.498	0.899	0.0	1.000	–
	Caucasian	4	CC vs. GG	0.208 (0.037–1.172)	0.075	0.357	0.0	1.000	–
			CG vs. GG	0.913 (0.599–1.391)	0.672	0.111	50.2	0.734	0.500
			CC + CG vs. GG	0.878 (0.317–2.428)	0.802	0.029	66.9	0.734	0.482
			CC vs. GG + CG	0.283 (0.047–1.722)	0.171	0.545	0.0	1.000	–
			C vs. G	0.869 (0.329–2.300)	0.778	0.028	66.9	0.734	0.518
	Mix (Brazilian)	2	CC vs. GG	0.890 (0.117–6.743)	0.910	0.651	0.0	1.000	–
			CG vs. GG	0.648 (0.101–4.170)	0.648	0.014	83.6	1.000	–
			CC + CG vs. GG	0.691 (0.102–4.690)	0.705	0.010	84.8	1.000	–
			CC vs. GG + CG	0.984 (0.126–7.706)	0.988	0.762	0.0	1.000	–
			C vs. G	0.769 (0.129–4.591)	0.774	0.012	84.0	1.000	–

A random effects model was used when *P*-value for heterogeneity test was < 0.1; otherwise, a fixed effects model was used, and values in bold were statistically significant at *P* < 0.05

*CHC* chronic hepatitis C; *CI* confidence interval, *HL* healthy, *OR* odds ratio, *SNP* single-nucleotide polymorphism; *P* (Q-test) *P*-value of Q-test for heterogeneity test

#### TGF-ß1–509C/T polymorphism (rs1800469) and chronic HCV infection

**Fig. 2:**
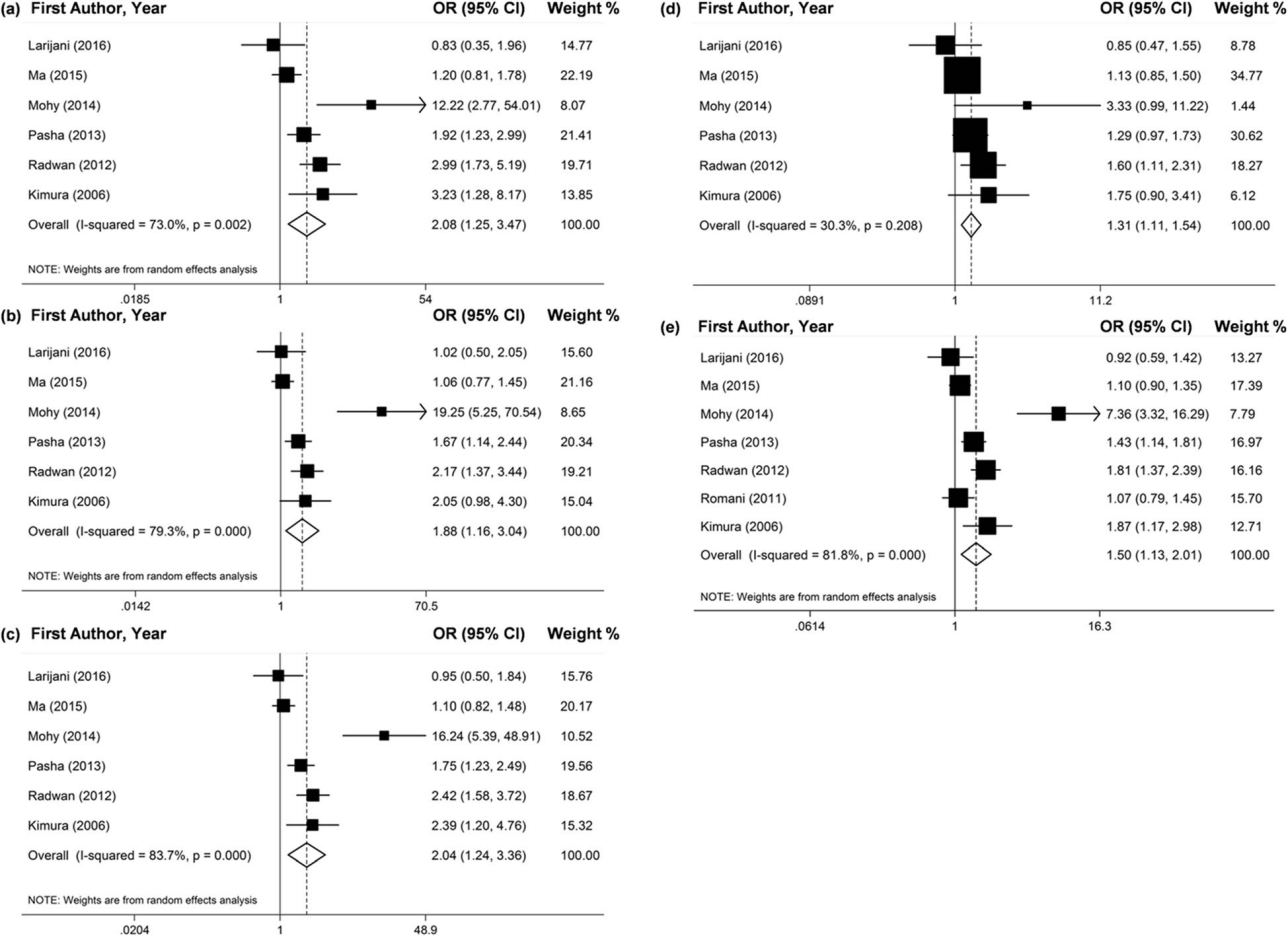
Forest plots of pooled OR with 95% CI for associations between *TGF-ß1*–509C/T polymorphism and the chronic HCV infection risk in total populations (a. TT vs. CC; b. TC vs. CC; c. TT + TC vs. CC; d. TT vs. TC + CC; e. T vs. C)

**Fig. 3:**
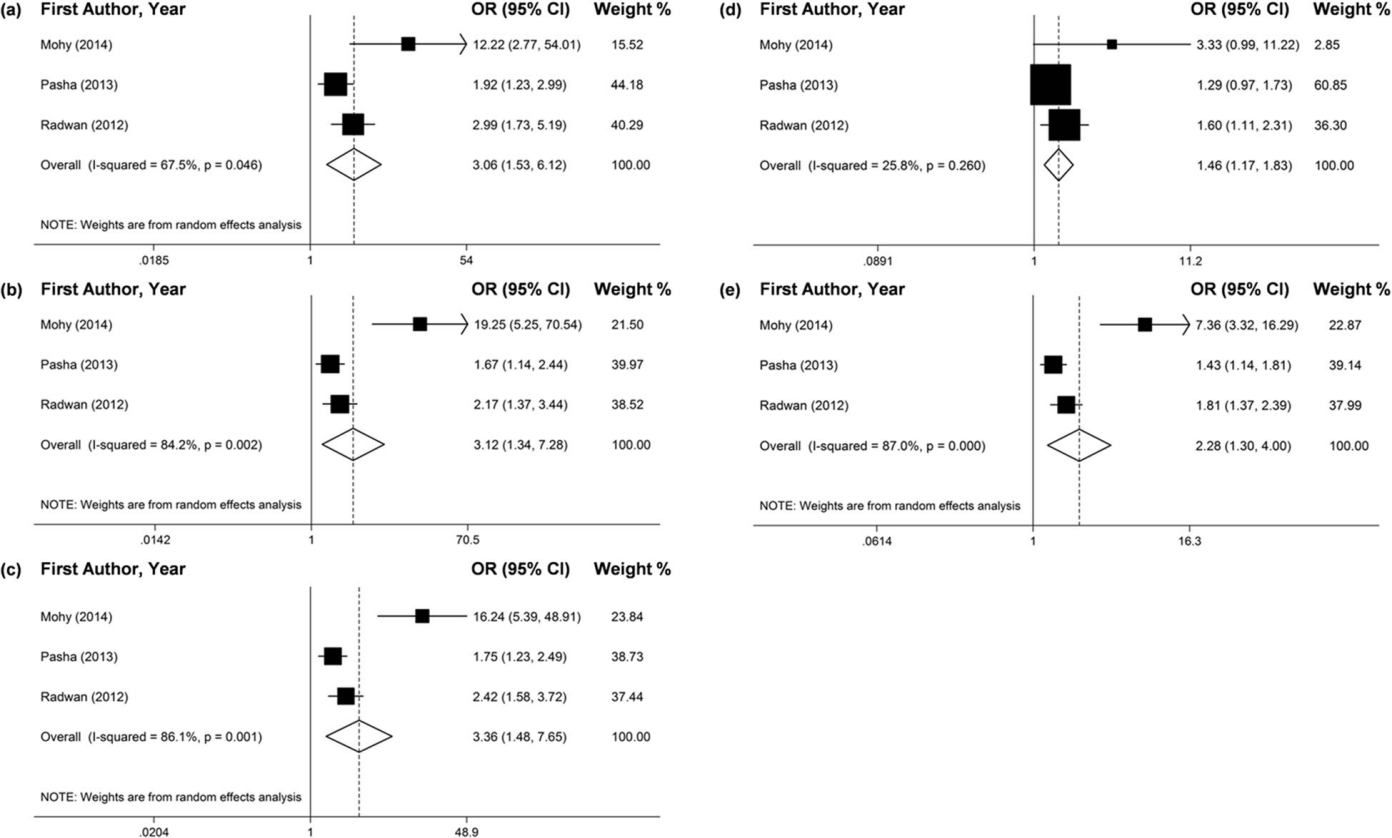
Forest plots of pooled OR with 95% CI for associations between *TGF-ß1*–509C/T polymorphism and the chronic HCV infection risk in the Egyptian population (a. TT vs. CC; b. TC vs. CC; c. TT + TC vs. CC; d. TT vs. TC + CC; e. T vs. C)

Seven studies investigated the connection between the *TGF-ß1*–509C/T polymorphism and the chronic HCV infection. Two of them didn’t satisfy the HWE rule by χ^2^ test [6,12], and another article just reported the allele distribution of T and C [17]. By the adaptive selection effect models, the effects of pooling included studies revealed that −509TT genotype and T allele may significantly increase the risk of chronic HCV infection in all genetic models (Fig. 2 and Table 2). In addition, the results of subgroup analyses by ethnicity presented that the polymorphism of -509C/T may significantly increase the risk of chronic HCV infection for Egyptians (Fig. 3 and Table 2).

**Fig. 4:**
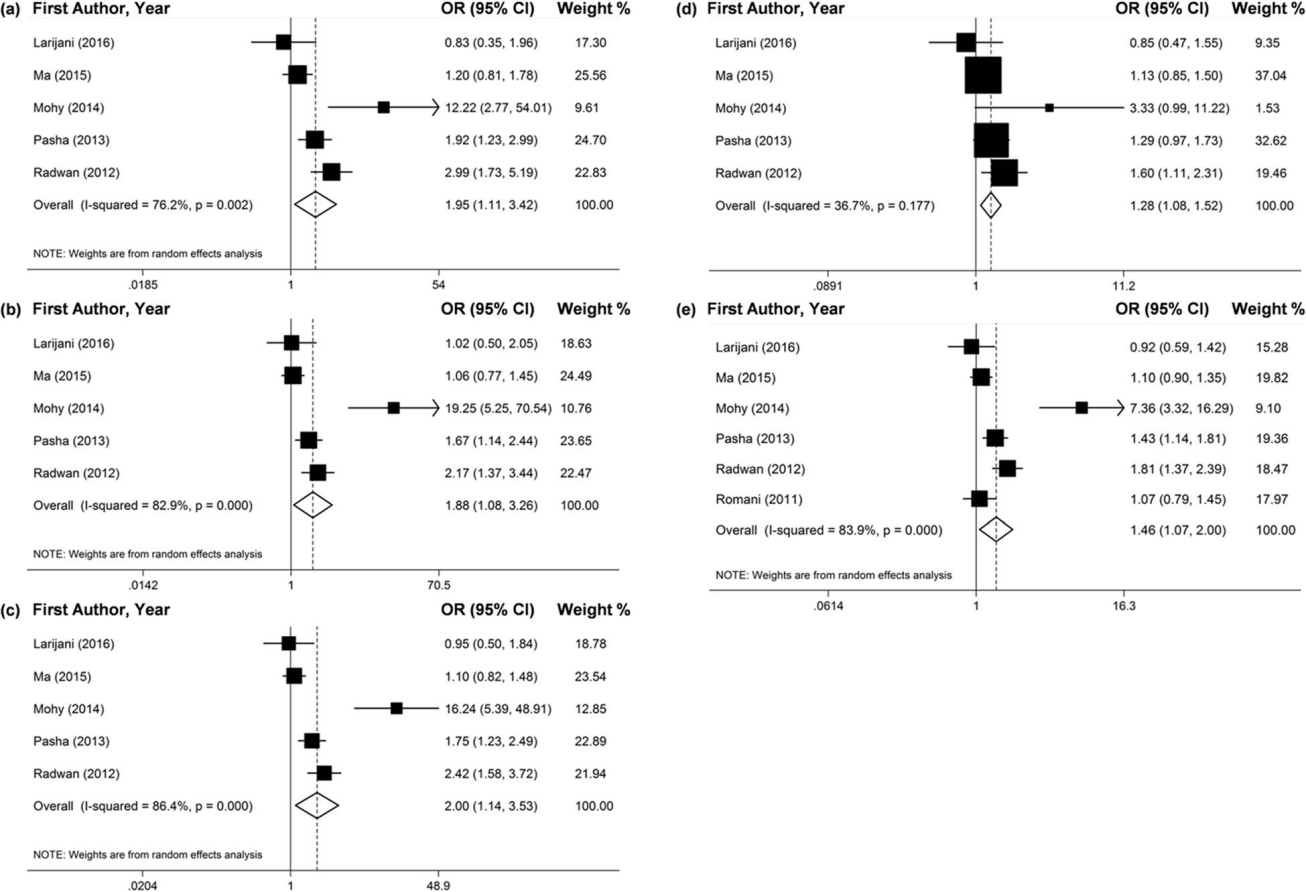
Forest plots of pooled OR with 95% CI for associations between *TGF-ß1*–509C/T polymorphism and the susceptibility to chronic HCV infection in the total populations (a. TT vs. CC; b. TC vs. CC; c. TT + TC vs. CC; d. TT vs. TC + CC; e. T vs. C)

The results of meta-analysis which contrasted the chronic HCV infected patients and healthy controls revealed that -509TT genotype and T allele promoted a higher risk of susceptibility to the chronic HCV infection in all gene models (Fig. 4 and Table 3). For the ethnicity subgroup analyses, more significant connection of -509TT genotype and T allele and chronic HCV infection was found in the Egyptian population (Fig. 3 and Table 3).

Since only one included study reported the comparison of chronic HCV infected cases and SR controls, we couldn’t conduct the pooling strategy (meta-analysis) to assess the association between the polymorphism of -509C/T and the spontaneous clearance of HCV [14].

**Fig. 5:**
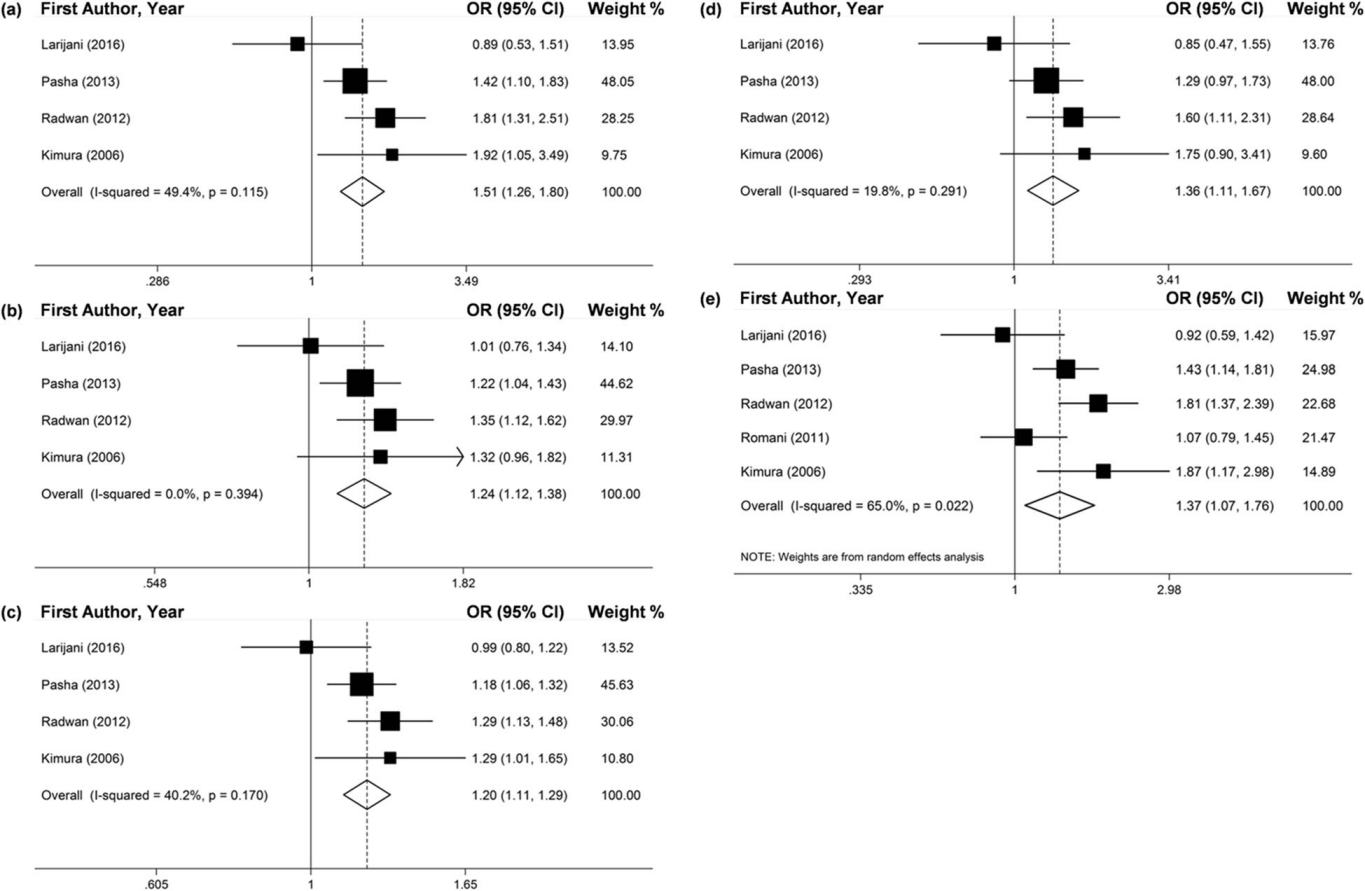
Forest plots of pooled OR with 95% CI for associations between *TGF-ß1*–509C/T polymorphism and the chronic HCV infection risk in total populations followed HWE(a. TT vs. CC; b. TC vs. CC; c. TT + TC vs. CC; d. TT vs. TC + CC; e. T vs. C)

**Fig. 6:**
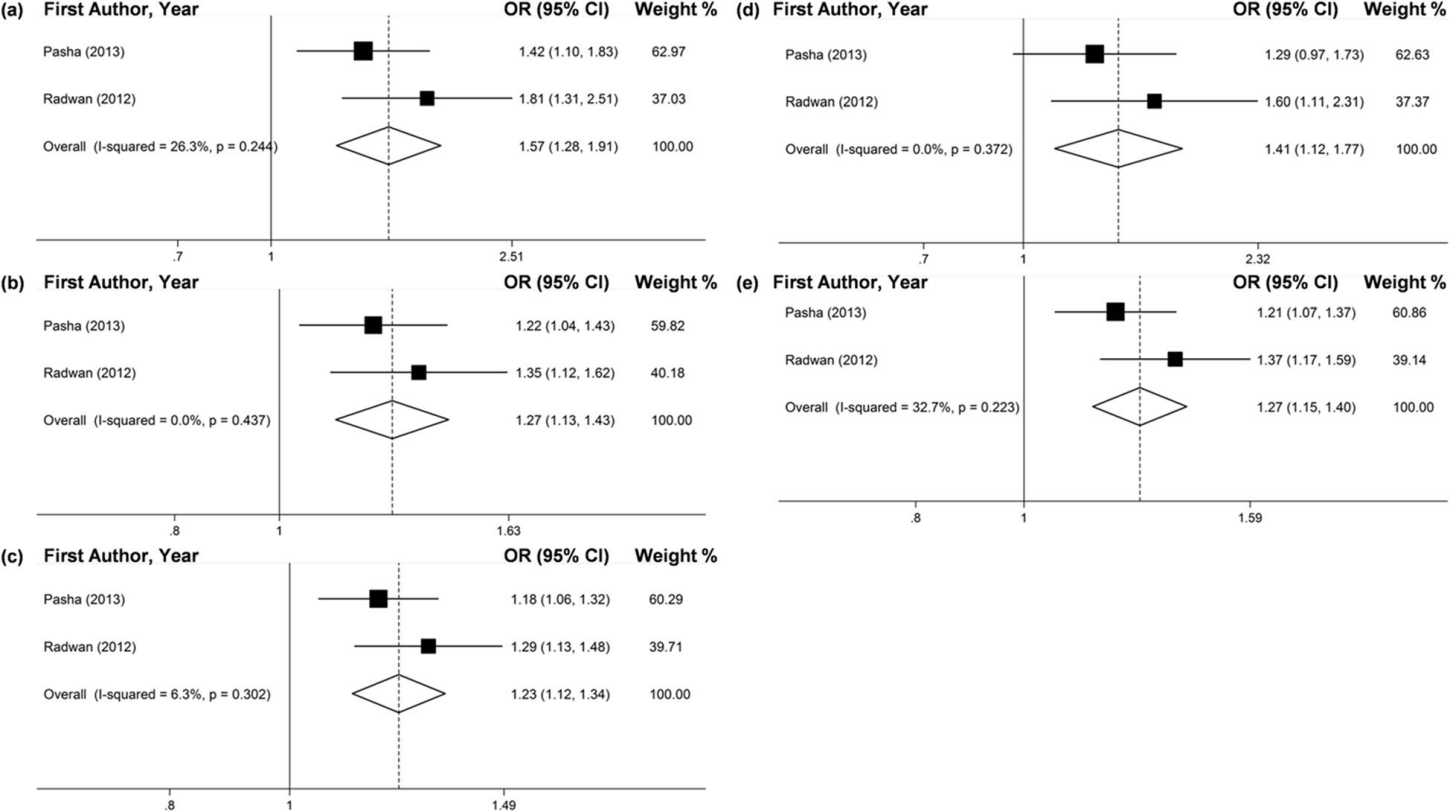
Forest plots of pooled OR with 95% CI for associations between *TGF-ß1*–509C/T polymorphism and the chronic HCV infection risk in the Egyptian population followed HWE (a. TT vs. CC; b. TC vs. CC; c. TT + TC vs. CC; d. TT vs. TC + CC; e. T vs. C)

**Fig. 7:**
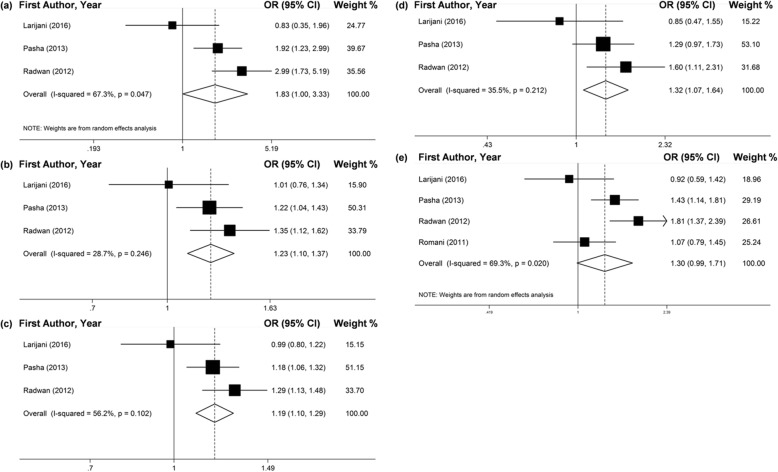
Forest plots of pooled OR with 95% CI for associations between *TGF-ß1*–509C/T polymorphism and the susceptibility to chronic HCV infection in the total populations followed HWE (a. TT vs. CC; b. TC vs. CC; c. TT + TC vs. CC; d. TT vs. TC + CC; e. T vs. C)

The related summary effects (ORs) rarely changed after we excluded the studies that didn’t follow the HWE (Figs. 5, 6 and 7 and Table 4).

**Table 4:** Main results of the meta-analysis of TGF-ß1 gene polymorphisms in populations following HWE

SNPs		No.of study	Gene model	OR(95%CI)	*P*	Heterogeneity text		Publication bias	
						*P*(Q-test)	*I*^2^(%)	Begg’s	Egger’s
-509	Total (HWE)	5	TT vs. CC	**1.507 (1.262–1.799)**	**0.000**	**0.115**	**49.4**	**1.000**	**0.400**
			TC vs. CC	**1.240 (1.117–1.377)**	**0.000**	**0.394**	**0.0**	**1.000**	**0.304**
			TT + TC vs. CC	**1.200 (1.113–1.295)**	**0.000**	**0.170**	**40.2**	**1.000**	**0.353**
			TT vs. CC + TC	1.141 (0.935–0.393)	0.193	0.504	0.0	1.000	0.563
			T vs. C	**1.374 (1.074–1.758)**	**0.011**	**0.022**	**65.0**	**0.806**	**0.403**
	Egyptian (HWE)	2	TT vs. CC	**1.567 (1.284–1.912)**	**0.000**	**0.244**	**26.3**	**1.000**	–
	(CHC-healthy)		TC vs. CC	**1.272 (1.128–1.434)**	**0.000**	**0.437**	**0.0**	**1.000**	–
			TT + TC vs. CC	**1.225 (1.124–1.336)**	**0.000**	**0.302**	**6.3**	**1.000**	–
			TT vs. CC + TC	**1.407 (1.120–1.768)**	**0.003**	**0.372**	**0.0**	**1.000**	–
			T vs. C	**1.270 (1.153–1.399)**	**0.000**	**0.223**	**32.7**	**1.000**	–
	Total (HWE)	4	TT vs. CC	**1.462 (1.215–1.760)**	**0.000**	**0.075**	**61.4**	**1.000**	**0.411**
	(CHC-healthy)		TC vs. CC	**1.230 (1.101–1.373)**	**0.000**	**0.246**	**28.7**	**1.000**	**0.355**
			TT + TC vs. CC	**1.189 (1.098–1.288)**	**0.000**	**0.102**	**56.2**	**1.000**	**0.384**
			TT vs. CC + TC	**1.323 (1.069–1.636)**	**0.010**	**0.212**	**35.5**	**1.000**	**0.427**
			T vs. C	1.300 (0.991–1.707)	0.058	0.020	69.3	0.308	0.228
codon 25	Total (HWE)	10	CC vs. GG	0.701 (0.417–1.180)	0.182	0.735	0.0	1.000	0.987
			CG vs. GG	0.974 (0.550–1.724)	0.927	0.002	67.5	0.602	0.775
			CC + CG vs. GG	0.956 (0.543–1.683)	0.875	0.001	68.5	0.754	0.769
			CC vs. GG + CG	0.766 (0.443–1.325)	0.341	0.896	0.0	1.000	0.924
			C vs. G	0.973 (0.626–1.512)	0.902	0.007	60.6	0.371	0.708
	Total (HWE)	8	CC vs. GG	0.696 (0.411–1.179)	0.178	0.596	0.0	0.806	0.913
	(CHC-healthy)		CG vs. GG	0.853 (0.474–1.533)	0.594	0.007	64.2	0.711	0.722
			CC + CG vs. GG	0.843 (0.468–1.518)	0.569	0.004	66.2	0.902	0.736
			CC vs. GG + CG	0.769 (0.441–1.342)	0.356	0.803	0.0	0.806	0.886
			C vs. G	0.864 (0.516–1.444)	0.576	0.008	63.4	0.711	0.723

A random effects model was used when *P*-value for heterogeneity test was < 0.1; otherwise, a fixed effects model was used, and values in bold were statistically significant at *P* < 0.05. –, no number

*CHC* chronic hepatitis C, *CI* confidence interval, *HWE* Hardy–Weinberg equilibrium, *OR* odds ratio, *SNP* single-nucleotide polymorphism, *P* (Q-test), *P*-value of Q-test for heterogeneity test

#### TGF-ß1 codon 10 T/C polymorphism (rs1982073) and chronic HCV infection

Nine studies investigated the connection between the polymorphism of codon 10 T/C and the chronic HCV infection. The summary results revealed no strong connections between the codon 10 T/C polymorphism and the risk of chronic HCV infection in total or subgroup analyses (Tables 2 and 3). Only one study compared the patients with the SR controls, therefore, we were unable to evaluate the association of this SNP with the spontaneous clearance of HCV [23].

#### TGF-ß1 codon 25G/C polymorphism (rs1800471) and chronic HCV infection

Eleven studies explored the role of the codon 25G/C polymorphism on the chronic HCV infection. One of them just reported the allele distribution of C and G [17]. Overall, the pooling estimates showed no significant association between the codon 25G/C polymorphism and chronic HCV infection in all comparison models of total or subgroup analyses (Tables 2 and 3).

We were able to compare the chronic HCV infected cases with SR controls in the contrast of C vs. G in two studies [17, 23], but the pooling estimates showed the codon 25G/C polymorphism was not associated with the spontaneous clearance of HCV (C vs. G: OR = 1.286, 95% CI = 0.768–2.153, *P* = 0.338).

### Sensitivity analysis

To test the stability of the summary effects model, we conducted the sensitive analysis by successively excluding single study. The summary effects were said stable if the pooling results rarely changed as the included studies successively were excluded. Take the analysis of -509C/T polymorphism in total control group as an example, when we successively excluded the studies with different data properties: Ma’s study which didn’t follow HWE; Mohy’s study whose sample size was the smallest one; and Pasha’s study whose sample size was the biggest one. The corresponding summary ORs were little altered (OR = 2.401, 95% CI = 1.350–4.270, *P* = 0.003; OR = 1.591, 95% CI = 1.106–2.290, *P* = 0.010; OR = 2.209, 95% CI = 1.130–4.320, *P* = 0.000, successively) (Additional file 5: Figure S1).

### Publication bias

The potential publication bias of studies for our meta-analysis conducted by the Begg’s funnel plot and the Egger’s test. The result indicated that publication bias of studies played a rare influence on our meta-analysis.

## Discussion

Millions of people are infected with HCV which can be considered as one of the most frequent infectious diseases. It is still not clear about the reason that caused the differences in the outcome of HCV-infected patients and how to effectually clear the HCV from human body. Lots of researchers worked on finding the risk factors which may explain the curative mechanism of HCV infection. Although multiple factors have been reported, the genetic factors are thought as relative radical solutions for the curative mechanism of HCV infection. Recently, several papers presented that the polymorphisms of *TGF-β1* may be responsible for HCV infection by affecting the expression and secretion of some cytokines. Since experiment data of individual study always shows imbalance of the structural which specifically performed as the difference in data size, ethnicities, regions and so on, the studies focusing on same subjects obtained controversial conclusions. These controversial conclusions were detrimental to clinical practice.

Through the comprehensive meta-analysis of the three SNPs locus (−509C/T, codon 10 T/C, and codon 25G/C) in *TGF-ß1* gene connecting with chronic HCV infection, we observed that -509C/T polymorphism might be a risk locus for chronic HCV infection, and the results of meta-analysis showed that this gene correlation were especially salient in Egyptians. However, the polymorphisms of codon 10 T/C or codon 25G/C exhibited no association with chronic HCV infection in total or subgroup analyses.

To further discriminate the effect of infection or clearance, we compared the chronic HCV infected patients with healthy controls or SR controls, respectively. Specifically, the summary results of our meta-analysis revealed that subjects with the genotype of -509TT and T allele have a about 2 and 3 fold higher stake of the susceptibility to chronic HCV infection in total and Egyptian populations, respectively. Moreover, the related summary effects (ORs) rarely changed after we excluded the studies that didn’t follow the HWE. A series of statistical tests which included the HWE test, sensitivity analysis and publication bias test guaranteed the related pooling ORs were stable, definite and statistically significant.

Several studies reported the polymorphism of codon 10 T/C was located at position + 29, relative the translational start site of *TGF-ß1* gene. The transition of T to C of the codon 10 may impact the export productivity of the newly synthesized protein [10, 11]. Wang et al suggested the codon 10C allele was likely to develop more severe fibrosis during chronic HCV infection [19]. And Vidigal et al reported the codon 10CC genotype was associated with the resistance to combined antiviral therapy in HCV infection [26]. However, in most other studies, the polymorphism of codon 10 T/C was not related to the chronic HCV infection [15, 16, 21–23, 30]. And our meta-analysis summary results of this SNP were consistent with the majority opinion. The deterministic correlation of codon 10 T/C polymorphism and chronic HCV infection needs more case-controls studies to confirm.

Another crucial SNP locus is codon 25G/C which may impact the production of TGF-β1. The transition of G to C may be correlated with the reduced level of TGF-β1 in vitro [27]. Pereira et al reported that the CHC patients have the higher frequency of codon 25G allele than healthy subjects [21]. Theoretically, it is plausible that subjects with the high TGF-β1 producer phenotype which is associated with the codon 25G allele present over suppression in the human immune response. This mechanism may result in the correlation of the polymorphism of codon 25G/C and chronic HCV infection. However, one previous meta-analysis demonstrated that the polymorphism of *TGF-β1* codon 25G/C wasn’t correlated with the chronic HCV infection [31]. In our proposed meta-analysis, the summary results were obtained by pooling the data from the latest and most complete studies and conducting the total analysis, subgroup analyses and the different comparison of controls analyses. Eventually, we didn’t find the connection between the polymorphism of *TGF-β1* codon 25G/C and the chronic HCV infection. Several factors may contribute to the discrepancy. First, the small size samples of included studies were insufficient to mining the deep connections of the certain genotype and certain clinical disease. Second, some impacted heterogeneity still existed, although we conducted subgroup analyses by the ethnicity and control groups as well as dropped the studies which did not satisfy the HWE rule to explore the sources of heterogeneity. Moreover, other factors such as other polymorphisms or viral factors of the chronic HCV infection may be involved. Notably, the codon 25 polymorphism has been revealed to be connected with the stage/degree of liver fibrosis in Caucasian HCV infected patients [32, 33]. A large-scale study is thereby required to confirm the genetic contribution to HCV infection or live fibrosis.

Due to the unique SR controls of related studies, we weren’t able to use meta-analysis to access the connection of the -509C/T or codon 10 T/C polymorphisms with the clearance of HCV. Moreover, the proposed meta-analysis results of two studies in the gene model of C vs. G showed that no significant association was found between the polymorphism of -25G/C and the spontaneous clearance of HCV. So deep researchs are warranted to accurately clarify the correlation of these SNPs and the spontaneous clearance of HCV.

Previous study reported high differences of these allelic frequencies from different ancestries [22]. Therefore, we conducted subgroup analyses by the ethnicity. The populations from Iran, Germany, American-Caucasian, and Ireland were classified as the Caucasians; the populations from Japan, Pakistan, and China were classified as the Asians; and the populations from Brazil, Morocco, and Mexico were classified as the mixed races populations. For the race of Egyptian, we searched for the relevant articles and consulted the geographers, but the results were inconsistent. In order to reduce the substantial genetic heterogeneity, we categorized the Egyptian as a separate subgroup.

For the purpose of accurately interpreting the results of this meta-analysis, several potential limitations should be declared. First, although we collected all published studies, the number of included study cases specific to each site was still limited, especially for the researches on the connection of spontaneous clearance of HCV. Randomized controlled clinical studies with larger sample sizes and multi-ethnic populations are required. Second, HCV infection and disease progression are influenced by multiple factors, and the potential gene-gene or gene-environment interactions should be conducted relative research. Finally, because some researches did not provide information such as gender, age and environmental factors, it is hard for us to study the effects of these factors on the connection of the polymorphisms of *TGF-ß1* gene with chronic HCV infection by a subgroup analysis.

## Conclusion

Taken together, our results suggested that the *TGF-ß1–*509 TT genotype and T allele were connected with a higher incidence of chronic HCV infection, and this connection was especially significant in Egyptians. In future studies, more researches with large scale samples and detail information will be required to enhance or correct the summary conclusions of the meta-analysis.

## Additional files

Additional file 1: **Table S1.** Detailed information of the *TGF-ß1*–509C/T in the studies associated with the chronic HCV infection included in the meta-analysis. (DOC 40 kb)

Additional file 2: **Table S2.** Detailed information of the *TGF-ß1* codon 10 T/C in the studies associated with the chronic HCV infection included in the meta-analysis. (DOC 44 kb)

Additional file 3: **Table S3.** Detailed information of the *TGF-ß1* codon 25 G/C in the studies associated with the chronic HCV infection included in the meta-analysis. (DOC 45 kb)

Additional file 4: **Table S4.** Scale for Quality Assessment for Identified Studies on TGF-ß1 gene polymorphisms and chronic HCV infection. (DOCX 15 kb)

Additional file 5: **Figure S1.** Sensitivity analysis of the -509C/T polymorphism in total populations (a. total populations; b. after excluding the Ma’s study; c. after excluding the Mohy’s study; d. after excluding the Pasha’s study). (TIF 1531 kb)

## Data Availability

Please contact the corresponding author for data requests.

## Abbreviations

AS: Asymptomatic carriers
CHC: Chronic hepatitis C
CI: Confidence interval
HCC: Hepatocellular carcinoma
HCV/HBV: Hepatitis C/B virus
HIV: Human immunodeficiency virus
HWE: Hardy–Weinberg equilibrium
IFN-γ: Interferon-gamma
IL: Interleukin
LC: Liver cirrhosis
NK: Natural killer
OR: Odds ratio
SNPs: Single-nucleotide polymorphisms
SR: Spontaneously recovered
TGF-ß: Transforming growth factor-beta
