# Supplementary material for: Association of TGF-ß1 polymorphisms and chronic hepatitis C infection: a Meta-analysis
Source: BMC Infect Dis. 2019 Aug 30;19:758. doi: 10.1186/s12879-019-4390-8 (PMC6716859; doi:10.1186/s12879-019-4390-8)
Supplement: Supplementary file 1 — Table S1. Detailed information of the TGF-ß1–509C/T in the studies associated with the chronic HCV infection included in the meta-analysis. (DOC 40 kb) [file 12879_2019_4390_MOESM1_ESM.doc]

| **Table S1.** TGF-ß1-509C/T polymorphism genotype distribution in cases and controls | | | | | | | | | | | | | |
| --- | --- | --- | --- | --- | --- | --- | --- | --- | --- | --- | --- | --- | --- |
| **First auther** | **Year** | **Genotype distribution** | | | | | | | | | | | **P for HWE** |
| **[Reference]** |  | **Case** | | | | |  | **Control** | | | | | **in control** |
|  |  | **TT** | **TC** | **CC** | **T** | **C** |  | **TT** | **TC** | **CC** | **T** | **C** | **(Y/N)** |
| Larijani [20] | 2016 | 17 | 43 | 29 | 77 | 101 |  | 17 | 35 | 24 | 69 | 83 | 0.536 (N) |
| Ma [13] | 2015 | 84 | 168 | 141 | 336 | 450 |  | 71 | 161 | 143 | 303 | 447 | 0.036 (N) |
| Mohy [6] | 2014 | 10 | 21 | 9 | 41 | 39 |  | 3 | 4 | 33 | 10 | 70 | 0.001 (N) |
| Pasha [14] | 2013 | 124 | 211 | 105 | 459 | 421 |  | 48 | 94 | 78 | 190 | 250 | 0.055 (Y) |
| Radwan [7] | 2012 | 84 | 138 | 58 | 306 | 254 |  | 30 | 68 | 62 | 128 | 192 | 0.147 (Y) |
| Romani [15] | 2011 |  |  |  | 178 | 150 |  |  |  |  | 178 | 160 |  |
| Kimura [26] | 2005 | 56 | 89 | 39 | 201 | 167 |  | 8 | 20 | 18 | 36 | 56 | 0.554 (Y) |
|  | | | | | | | | | | | | | |

Table S1 Detailed information of the *TGF-ß1*-509C/T in the studies associated with the chronic HCV infection included in the meta-analysis.
