# Supplementary material for: Association of TGF-ß1 polymorphisms and chronic hepatitis C infection: a Meta-analysis
Source: BMC Infect Dis. 2019 Aug 30;19:758. doi: 10.1186/s12879-019-4390-8 (PMC6716859; doi:10.1186/s12879-019-4390-8)
Supplement: Supplementary file 2 — Table S2. Detailed information of the TGF-ß1 codon 10 T/C in the studies associated with the chronic HCV infection included in the meta-analysis. (DOC 44 kb) [file 12879_2019_4390_MOESM2_ESM.doc]

| **Table S2.** TGF-ß1 codon 10T/C polymorphism genotype distribution in cases and controls | | | | | | | | | | | | | |
| --- | --- | --- | --- | --- | --- | --- | --- | --- | --- | --- | --- | --- | --- |
| **First auther** | **Year** | **Genotype distribution** | | | | | | | | | | | **P for HWE** |
| **[Reference]** |  | **Case** | | | | |  | **Control** | | | | | **in control** |
|  |  | **CC** | **TC** | **TT** | **C** | **T** |  | **CC** | **TC** | **TT** | **C** | **T** | **(Y/N)** |
| Imran [21] | 2014 | 20 | 74 | 46 | 114 | 166 |  | 20 | 66 | 34 | 106 | 134 | 0.207 (Y) |
| Romani [15] | 2011 | 35 | 85 | 49 | 155 | 183 |  | 33 | 81 | 50 | 147 | 181 | 0.985 (Y) |
| Pereira [25] | 2008 | 26 | 65 | 37 | 117 | 139 |  | 24 | 49 | 21 | 97 | 91 | 0.672 (Y) |
| Wang [27] | 2005 | 23 | 100 | 87 | 146 | 274 |  | 4 | 27 | 19 | 35 | 65 | 0.186 (Y) |
| Zein [19] | 2004 | 3 | 15 | 6 | 21 | 27 |  | 11 | 22 | 12 | 44 | 46 | 0.884 (Y) |
| Zein2 [19] | 2004 | 4 | 17 | 10 | 25 | 37 |  | 3 | 21 | 12 | 27 | 45 | 0.142 (Y) |
| Suzuki [27] | 2003 | 66 | 84 | 56 | 216 | 196 |  | 21 | 52 | 28 | 94 | 108 | 0.728 (Y) |
| Barrett [29] | 2003 | 9 | 44 | 39 | 62 | 122 |  | 14 | 26 | 26 | 54 | 78 | 0.132 (Y) |
| Vidigal [30] | 2002 | 13 | 38 | 29 | 64 | 96 |  | 4 | 21 | 12 | 29 | 45 | 0.246 (Y) |

Table S2 Detailed information of the *TGF-ß1* codon 10 T/C in the studies associated with the chronic HCV infection included in the meta-analysis.
