# Supplementary material for: Association of TGF-ß1 polymorphisms and chronic hepatitis C infection: a Meta-analysis
Source: BMC Infect Dis. 2019 Aug 30;19:758. doi: 10.1186/s12879-019-4390-8 (PMC6716859; doi:10.1186/s12879-019-4390-8)
Supplement: Supplementary file 3 — Table S3. Detailed information of the TGF-ß1 codon 25 G/C in the studies associated with the chronic HCV infection included in the meta-analysis. (DOC 45 kb) [file 12879_2019_4390_MOESM3_ESM.doc]

| **Table S3.** TGF-ß1 codon 25G/C polymorphism genotype distribution in cases and controls | | | | | | | | | | | | | |
| --- | --- | --- | --- | --- | --- | --- | --- | --- | --- | --- | --- | --- | --- |
| **First auther** | **Year** | **Genotype distribution** | | | | | | | | | | | **P for HWE** |
| **[Reference]** |  | **Case** | | | | |  | **Control** | | | | | **in control** |
|  |  | **CC** | **GC** | **GG** | **C** | **G** |  | **CC** | **GC** | **GG** | **C** | **G** | **(Y/N)** |
| Imran [21] | 2014 | 18 | 53 | 69 | 89 | 191 |  | 17 | 48 | 55 | 82 | 158 | 0.225 (Y) |
| Rebbani [22] | 2014 |  |  |  | 11 | 77 |  |  |  |  | 7 | 96 |  |
| Romani [15] | 2011 | 1 | 18 | 145 | 20 | 308 |  | 2 | 16 | 151 | 20 | 318 | 0.052 (Y) |
| Pereira [25] | 2008 | 1 | 14 | 113 | 16 | 240 |  | 1 | 29 | 64 | 31 | 157 | 0.244 (Y) |
| Armenda´riz-Borunda [23] | 2008 | 0 | 0 | 13 | 0 | 26 |  | 6 | 13 | 11 | 25 | 35 | 0.552 (Y) |
| Fang [24] | 2008 | 3 | 9 | 73 | 15 | 155 |  | 3 | 15 | 88 | 21 | 191 | 0.033 (N) |
| Wang [27] | 2005 | 0 | 32 | 178 | 32 | 388 |  | 0 | 8 | 42 | 8 | 92 | 0.539 (Y) |
| Zein [19] | 2004 | 0 | 3 | 21 | 3 | 45 |  | 0 | 4 | 41 | 4 | 86 | 0.755 (Y) |
| Zein2 [19] | 2004 | 0 | 6 | 25 | 6 | 56 |  | 0 | 3 | 33 | 3 | 69 | 0.794 (Y) |
| Barrett [28] | 2003 | 0 | 14 | 18 | 14 | 50 |  | 1 | 16 | 49 | 18 | 114 | 0.812 (Y) |
| Vidigal [30] | 2002 | 1 | 11 | 68 | 13 | 147 |  | 0 | 3 | 34 | 3 | 71 | 0.797 (Y) |

Table S3 Detailed information of the *TGF-ß1* codon 25 G/C in the studies associated with the chronic HCV infection included in the meta-analysis.
