# Supplementary material for: Association of TGF-ß1 polymorphisms and chronic hepatitis C infection: a Meta-analysis
Source: BMC Infect Dis. 2019 Aug 30;19:758. doi: 10.1186/s12879-019-4390-8 (PMC6716859; doi:10.1186/s12879-019-4390-8)
Supplement: Supplementary file 4 — Table S4. Scale for Quality Assessment for Identified Studies on TGF-ß1 gene polymorphisms and chronic HCV infection. (DOCX 15 kb) [file 12879_2019_4390_MOESM4_ESM.docx]

| **Criteria** | **Score** |
| --- | --- |
| 1. Representativeness of cases |  |
| Diagnosed criteria to acknowledged criteria | 2 |
| Mentioned the diagnosed criteria but not specifically described | 1 |
| Not described | 0 |
| 1. Source of controls |  |
| Population or community based | 3 |
| Hospital-based HCV-free controls | 2 |
| Healthy volunteers without total description | 1 |
| HCV-free controls with related diseases | 0.5 |
| Not described | 0 |
| 1. Sample size |  |
| >400 | 2 |
| 200-400 | 1 |
| <200 | 0 |
| 1. Quality control of genotyping methods |  |
| Repetition of partial/total tested samples with a different method | 2 |
| Repetition of partial/total tested samples with the same method | 1 |
| Not described | 0 |
| 5.Hardy-Weinberg equilibrium (HWE) |  |
| Hardy-Weinberg equilibrium in control subjects | 1 |
| Hardy-Weinberg disequilibrium in control subjects | 0 |

**Supplementary Table S4. Scale for Quality Assessment for Identified Studies on TGF-ß1 gene polymorphisms and chronic HCV infection.**
