# Supplementary figures and images for: Association of TGF-ß1 polymorphisms and chronic hepatitis C infection: a Meta-analysis
Source: BMC Infect Dis. 2019 Aug 30;19:758. doi: 10.1186/s12879-019-4390-8 (PMC6716859; doi:10.1186/s12879-019-4390-8)

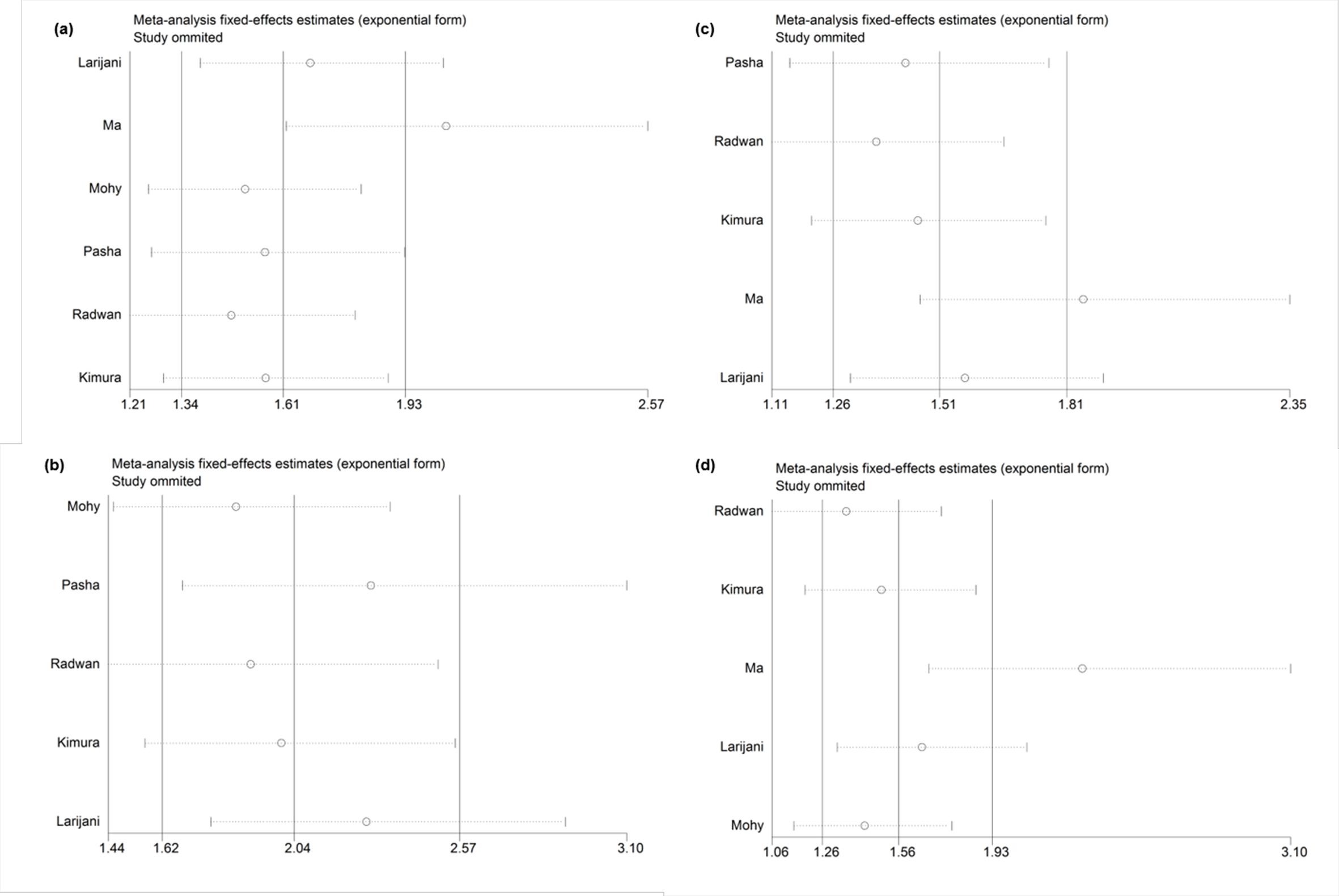

Supplement: Supplementary file 5 — Figure S1. Sensitivity analysis of the -509C/T polymorphism in total populations (a. total populations; b. after excluding the Ma’s study; c. after excluding the Mohy’s study; d. after excluding the Pasha’s study). (TIF 1531 kb) [file 12879_2019_4390_MOESM5_ESM.tif]
